# Development of a Hybrid Nanoprobe for Triple-Modality MR/SPECT/Optical Fluorescence Imaging

**DOI:** 10.3390/diagnostics4010013

**Published:** 2014-03-10

**Authors:** Renata Madru, Pontus Svenmarker, Christian Ingvar, Freddy Ståhlberg, Stefan-Andersson Engels, Linda Knutsson, Sven-Erik Strand

**Affiliations:** 1 Department of Medical Radiation Physics, Lund University, Barngatan 2, 221 85 Lund, Sweden; E-Mails: Freddy.Stahlberg@med.lu.se (F.S.); Linda.Knutsson@med.lu.se (L.K.); Sven-Erik.Strand@med.lu.se (S.-E.S.); 2 Department of Physics, Lund University, Professorsgatan 1, 223 63 Lund, Sweden; E-Mails: pontus.svenmarker@fysik.lth.se (P.S.); stefan.andersson-engels@fysik.lth.se (S.-A.E.); 3 Department of Surgery, Skane University Hospital, Entrégatan 7, 221 85 Lund, Sweden; E-Mail: Christian.Ingvar@med.lu.se; 4 Lund University Bioimaging Center (LBIC), Kliniggatan 32, 222 42 Lund, Sweden; 5 Department of Radiology, Skane University Hospital, Entrégatan 7, 221 85 Lund, Sweden

**Keywords:** SPION, SPECT, optical, fluorescence imaging, magnetic resonance imaging, MR, SLN

## Abstract

Hybrid clinical imaging is an emerging technology, which improves disease diagnosis by combining already existing technologies. With the combination of high-resolution morphological imaging, *i.e.*, MRI/CT, and high-sensitive molecular detection offered by SPECT/PET/Optical, physicians can detect disease progression at an early stage and design patient-specific treatments. To fully exploit the possibilities of hybrid imaging a hybrid probe compatible with each imaging technology is required. Here, we present a hybrid nanoprobe for triple modality MR/SPECT/Fluorescence imaging. Our imaging agent is comprised of superparamagnetic iron oxide nanoparticles (SPIONs), labeled with ^99m^Tc and an Alexa fluorophore (AF), together forming ^99m^Tc-AF-SPIONs. The agent was stable in human serum, and, after subcutaneous injection in the hind paw of Wistar rats, showed to be highly specific by accumulating in the sentinel lymph node. All three modalities clearly visualized the imaging agent. Our results show that a single imaging agent can be used for hybrid imaging. The use of a single hybrid contrast agent permits simultaneous hybrid imaging and, more conventionally, allow for single modality imaging at different time points. For example, a hybrid contrast agent enables pre-operative planning, intra-operative guidance, and post-operative evaluation with the same contrast agent.

## 1. Introduction

Traditional emission tomography and radiological imaging technologies provide valuable physiological, metabolic and morphological information for diagnosing of disease. A single imaging modality cannot, by itself, give complete information of disease and the underlying molecular aberrations causing it. A combination of modalities may give the necessary information that physicians need to detect disease progression at an early stage, design patient-specific treatments, and monitor *in vivo* post-therapeutic effects for therapy optimization [1]. For these reasons, improved multimodality imaging with molecular sensitivity could help pave the way for personalized medicine [2,3,4]. 

Multimodality imaging combines two or more imaging technologies in order to increase the diagnostic potential and to overcome the limitations offered by each single modality. A high-resolution imaging technology, providing morphological information, is typically combined with a high-sensitivity imaging technology sensing molecular aberrations. Examples of such systems include SPECT/CT, PET/CT, PET/MR, and MR/optical imaging modalities [5,6,7,8]. Advances in the development of SPECT/MR scanners and their distinct advantages have previously been reported [9,10]. SPECT and PET are highly sensitive but are limited by poor spatial resolution and lack of morphological information. A combination of SPECT or PET with CT imaging, which has the ability to acquire complementary whole body, high-resolution morphological images, has rapidly evolved as a new golden standard technique within oncology [11,12,13]. MR imaging, on the other hand, provides non-invasive, high-resolution functional and morphological images with better soft tissue contrast than CT. Its drawbacks are longer imaging time and the low sensitivity to molecular targets. Combining PET/SPECT or optical imaging with MR technology will enable a unique feature for detection and monitoring of diseases [14,15]. Emerging optical imaging technologies, such as fluorescence, bioluminescence and Cerenkov luminescence are fast (images can be acquired in <1 min), inexpensive and easy to implement in a clinical setting [16,17]. In order to fully exploit the advantages of such multimodality technologies, multimodal imaging agents are warranted. 

Superparamagnetic iron oxide nanoparticles (SPIONs) possess unique properties that make them well-suited for multimodality molecular imaging [18]. Initially, SPIONs were introduced as MR contrast agents and clinically approved for visualization of liver metastasis (Resovist^®^), angiography, and sentinel node mapping (Feridex^®^, Combidex^®^). The large surface to volume ratio also enables addition of different ligands for active targeting of, *i.e.*, immune cells, angiogenesis or plaque [19,20,21,22]. Furthermore, SPIONs show a great potential for *in vivo* stem cell tracking and monitoring gene delivery [23,24]. Labeled with radionuclides, SPIONs can be used for combined PET/MR or SPECT/MR imaging [25,26,27,28]. 

In our previous study, we suggested to use radiolabeled iron oxide nanoparticles (^99m^Tc-SPIONs) for early detection/staging of cancer by SPECT and MR [28]. Herein, we improved the properties of the ^99m^Tc-SPIONs by labeling them with a fluorescent dye, and not only ^99m^Tc and SPIONS for SPECT/MR, but also for optical fluorescence imaging. This triple-modality molecular imaging agent identifies the target by whole body SPECT and MR imaging, and highlight the target intra-operatively by fluorescence imaging. The radiolabeled and fluorescent SPIONs can also be visualized by microscopy during *ex vivo* pathological evaluation and the molecular targets can be quantified, e.g., by autoradiography. Here, we demonstrate that the attached dye does not affect the size and *in vivo* stability of the nanoparticles. The targeting ability of SLNs and biodistribution of the agent is demonstrated in Wistar rats. 

## 2. Experimental Section

### 2.1.SPIONs

The SPIONs used in this study have been described elsewhere [28]. Briefly, the SPIONs consist of a monodispersed superparamagnetic iron oxide core (Fe_3_O_4_) coated with polyethylene glycol and including primary amino groups. The coating prevents aggregation and enables functionalization. 

### 2.2.Labeling of SPIONs with Fluorescent Dye and Radioactivity

The SPIONs (0.5 mL, 3.4 mg/mL Fe) were labeled with the amino-reactive Alexa Fluor 647 (AF) carboxylic acid dye (Molecular probes, Life Technologies) by succinimidyl (NHS) ester coupling. The dye (10 mg) was dissolved in DMSO (1 mL) and incubated with the SPIONs for 2 h. The AF–SPIONs were washed three times with saline (0.9% v/v) and filtered after each wash by using a MACS MS column (Miltenyi Biotech, Germany) and a strong cylindrical magnet. When the magnet was attached to the column, SPIONs got trapped in the filter while the fluid ran through. By removing the magnet, the nanoparticles could be eluted with saline. 

The remaining free amino groups on the SPIONs surface were used to label the nanoparticles with ^99m^Tc. ^99m^Tc-pertechnetate (^99m^TcO_4_^−^) was obtained from a ^99^Mo/^99m^Tc-generator (DRN 4329 Ultratechnekow FM). The radiolabeling followed a previously developed protocol [28] and was slightly modified to obtain the highest labeling efficiency at pH 4–4.2. The protocol stated that stannous chloride (10 mg) was to be dissolved in 10 mL sterile water and stirred for 1 min. A part of the solution (0.5 mL) was passed through a 22 µm Millipore filter and added to the ^99m^TcO_4_^−^ (278 MBq, 1 mL), which was kept in a vacuumcollecting vial. The hydrogen ion activity was adjusted to pH 4 by adding NaOH (1 M, ~20 µL), after which, AF–SPIONs were added (0.1 mL) and gently shaken for 30 s. The mixture was incubated for 1 h at room temperature. The fluorescence and radiolabeled SPIONs (^99m^Tc-AF–SPIONs) were filtered through MACS columns as described previously and eluted in saline (0.9% v/v, ~0.25 mL). 

### 2.3. Quality Control

The radiolabeling yield was measured with instant-thin layer chromatography (ITLC). A small volume (0.8 µL) of reaction mixture was dropped on silica-gel impregnated glass fiber sheets (5 × 10 cm ITLC-SG, Pall ITLC Media, Pall Life Sciences) and aqueous methanol (85% v/v) was used as mobile phase for developing. The sheets were dried and cut in two strips, of 3 cm and 7 cm. The two segments were compared for radioactivity, as measured by a NaI(Tl) well type detector (1282 CompuGamma CS, LKB Wallac). 

The reaction mixture, containing ^99m^Tc-AF-SPIONs, was filtered and pre-concentrated using MACS MS columns (Miltenyi Biotech, Germany). The radioactivity of the buffer solution was compared to the radioactivity of the filtered ^99m^Tc-AF-SPIONs. 

Two ^99m^Tc-AF-SPION mixtures (10 µL) were diluted in human serum (500 µL) and incubated for 24 h at pH 7 in room temperature. The ITLC method was then used to measure any release of free ^99m^Tc. SPIONs and ^99m^Tc-AF-SPIONs were imaged with transmission electron microscopy (TEM, Philips CM 10 System, USA) using 400 mesh carbon grids to measure the size of the nanoparticles and to evaluate possible aggregation. 

### 2.4. Animal Studies

To show the *in vivo* feasibility for imaging, two white Wistar rats weighing 250–300 g were used. The experiments were completed in compliance with local and national regulations approved by the Local Ethics Committee for Animal Research. The animals were slightly anesthetized with isoflurane and injected subcutaneously in the right hind paw, with ^99m^Tc-AF-SPIONs (0.07–0.1 mL) dispersed in physiological saline buffer (~40 MBq, ~0.3 mg Fe). The animals returned to their cages and, 5 h post injection, imaged with MR, SPECT and Optical modalities. To assess the activity distribution the animals were sacrificed and tissues/organs were measured in the NaI(Tl) well type detector (1282 CompuGamma CS, LKB Wallac).

#### 2.4.1. MR Imaging

The animals were imaged with a 3T-MR system (Philips Achieva) equipped with a Sense-Flex-M coil. The images were acquired using Spin Echo (SE) and 3D Gradient Echo (GRE) pulse sequences with the following parameters. SE: TE/TR 80/3000 ms; FOV 100 mm; voxel size 0.5 × 0.5 × 1.3 mm^3^; flip angle 90° and number of average 10. GRE: TE/TR 14.6/2000 ms; voxel size 0.8 × 0.8 × 1.3 mm^3^; flip angle 90° and number of averages 10.

#### 2.4.2. SPECT Imaging

A scintillation camera (GE Hawkeye; GE Healthcare) captured anterior and posterior images in 15 min. The camera was equipped with a low energy, high-resolution parallel-hole collimator. SPECT/CT projections were acquired for 1 h, at 60 s per projection, with a 20% energy window centered at 140 keV.

#### 2.4.3. Optical Imaging

The animals were shaved prior to imaging in an effort to minimize autofluorescence. Imaging of the animals was performed in epifluorescence mode. Laser induced fluorescence of the ^99m^Tc-AF-SPIONs was accomplished by a Helium-Neon laser (633 nm, 5 mW). The laser source illuminated the animal with a slightly divergent beam with a spot size of approximately 2 cm^2^. The illumination spot was raster-scanned in an overlapping pattern across the region of interest, and one image was acquired for each illumination position. An air-cooled charge coupled device camera (Andor iXon DU-897) captured the image after having passed through a combination of a long-pass filter (Schott, RG665) and a dielectric band-pass filter centered at 670 nm. All images were background subtracted, subjected to a threshold, and summed to give the resulting image. The resulting image was overlaid a photograph of the animal to give an anatomical reference.

### 2.5. Biodistribution

The animals were dissected; the lymph nodes, kidneys, spleen, liver, and the hind paw were weighed and measured for activity in the NaI(Tl) well-type detector (1282 CompuGamma CS; LKB Wallac). The radioactivity measurement was corrected for background and decay. The uptake of the ^99m^Tc-AF-SPIONs within the dissected organs was expressed in % of injected activity (% IA/g). 

The lymph nodes were frozen, cut in 100 µm and 20 µm thick frozen coronal sections using a cryostat (Microme, Vacutome HM 500 OM; Microme International) and mounted on microscope slides and air dried at room temperature. The 100 µm sections were imaged and analyzed by digital autoradiography (DARG) with a silicon strip detector (Biomolex 700 Imager; Biomolex AS). Each second cryo-slice was hematoxilyn and eosin (H&E) stained and used as a reference for the DARG images. The 20 µm sections were DAPI stained and used for microscopy analysis (Zeiss Axiovert 200M). 

## 3. Results and Discussion

Advances in development of hybrid imaging technologies have created an enormous interest in the design and preparation of novel multimodality imaging agents [29,30,31]. Among these imaging agents the nuclear/MR and MR/optical imaging probes are the most successful combinations due to its high degree of complementarity. 

In this work we propose combining information from MR, nuclear and optical images in order to have the full scale from whole-body imaging down to cellular and molecular imaging with use of one single agent. The major advantages to work with SPIONs are the easy production of the spherical core within narrow size range distribution, the simple coating procedure and the low *in vivo* toxicity. As the SPIONs are small in size and have a large area to volume ratio, a single nanoparticle can be labeled with a large number of radionuclides, fluorophores, and/or targeting ligands. In addition, polyethylene glycol coated SPIONs circulate in the blood pool/vasculature for a longer period of time compared with the low molecular weight gadolinium-based MR contrast imaging agents [32]. The labeling of SPIONs with a fluorescent dye and radionuclides may change the nanoparticles surface, physical and/or chemical properties. The final size of the ^99m^Tc-AF-SPIONs is crucial for targeting because it may influence the pharmacokinetic and biodistribution of the agent. 

### 3.1. Labeling of the SPIONs with Fluorescent Dye and ^99m^Tc

Despite the low cost of optical instrumentation and easy implementation for intra-operative surgery, there are only two fluorescent dyes (ICG and fluorescein sodium) approved by the FDA [33]. In basic research and preclinical imaging, the most commonly used fluorophores are those in the range of NIR [34]. The long excitation and emission wavelengths can penetrate deeper in tissue and give less autofluorescence. Cyanine dye Cy5 and Alexa Fluor (650 nm excitation, 670–680 nm emission) are almost as good at penetrating tissue as true NIR radiation. In addition, they are very stable, and can be visualized with standard light microscopy for pathological evaluation of samples and have easy conjugation chemistry. In this study, a simple and highly efficient conjugation protocol was developed for labeling SPIONs with AF. The ratio of fluorophore molecules to nanoparticles was calculated to be 300:1, leaving more than 200 free binding sites for labeling SPIONs with ^99m^Tc in order to evaluate the biodistribution of the agent *in vivo*. ^99m^Tc is a generator produced isotope, highly available and the physical properties such as half-life and emitted energy is well-suited for imaging and quantification purposes. 

### 3.2. Quality Control

The radiolabeling yield of the ^99m^Tc-AF-SPIONs, determined by ITLC, was 99%. ^99m^Tc-AF-SPIONs remained at the origin whereas free ^99m^Tc moved with the solvent front (Rf 1.0). Control measurements with ^99m^Tc-pertechnetate have been performed to validate the method. Additionally, the high radiolabeling efficiency was confirmed by magnetic separation and found to be 99.7%. 

**Figure 1:**
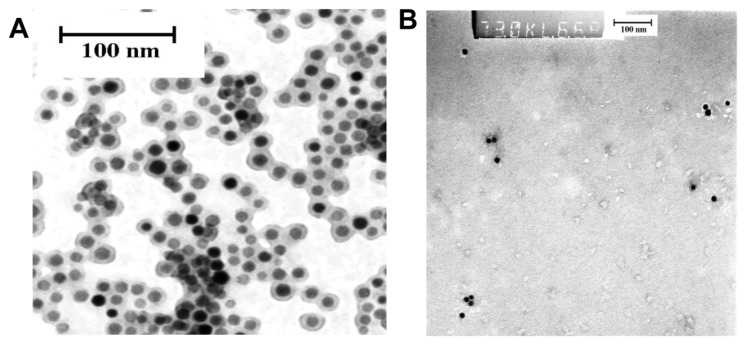
Transmission electron microscopy images of nanoparticles. (**a**) SPIONs in 0.9% saline buffer, pH 7 and (**b**) ^99m^Tc-AF-SPIONs after incubation in human serum at room temperature. The images demonstrate that the size of the nanoparticles is not affected by the labeling procedure including both radionuclides and fluorescent dye. No aggregations of the particles have been observed after incubation of ^99m^Tc-AF-SPIONs in human serum.

^99m^Tc-AF-SPIONs incubated in serum showed a good dispersion and was found to be stable. After 24 h incubation of the ^99m^Tc-AF-SPIONs, the amount of free ^99m^Tc in human serum was less than 3%, analyzed with the ITLC method. The stability of the ^99m^Tc-AF-SPIONs was also confirmed by the TEM images as demonstrated in Figure 1. The TEM images show that the size of the ^99m^Tc-AF-SPIONs is not affected by the labeling procedure; the spherical shape of the iron oxide core can be easily recognized and the surrounding polyethylene coating, which was shown to be intact. Both unlabeled (Figure 1A) and ^99m^Tc-AF SPIONs (Figure 1B) show a mean diameter of 11 nm ± 2 nm and no aggregation could be observed. Dynamic Light Scattering (DLS) is another method that can be used to measure the size of the nanoparticles as we presented in our previous work [28]. In this study DLS could not be applied because the fluorophores attached to the SPIONs have similar emission wave lengths as the laser used in the instrument and the size measurements may be corrupted. However, the attached fluorophores consists of very small molecules and have little effect of the overall size of the SPIONs. On the other hand, it can affect the stability (SPIONs can aggregate because the charge of the nanoparticles may change) and, therefore, we used TEM in this study.

### 3.3. Animal Studies

#### 3.3.1. MR Imaging

^99m^Tc-AF-SPIONs accumulated in lymph nodes, shortens the T2 relaxation of the spins and introduce local inhomogeneity in the magnetic field which shortens the T2* relaxation; consequently the signal intensity in tissue decrease providing a “negative contrast enhancement”. 

**Figure 2:**
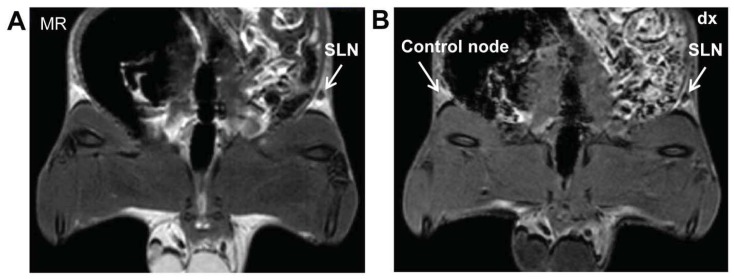
Representative coronal MR images of white Wistar rat injected subcutaneously with ^99m^Tc-AF-SPIONs in the right hind paw. Accumulation of the ^99m^Tc-AF-SPIONs in SLN can clearly visualized using (**a**) SE and (**b**) GRE sequences (white arrows).

The SLN could easily be recognized with both SE and GRE sequences as shown in Figure 2. The lymph nodes are embedded in fat tissue; the shape of the nodes can easily be distinguished due to the injected ^99m^Tc-AF-SPIONs. The lymph nodes on the contralateral sides have been used as control. However, without SPIONs the contrast between the lymph node and surrounding tissue is very low. The amount of the contrast agent injected shown to be well optimized, and no “blooming” artifacts were recognized in the images. 

#### 3.3.2. SPECT Imaging

**Figure 3:**
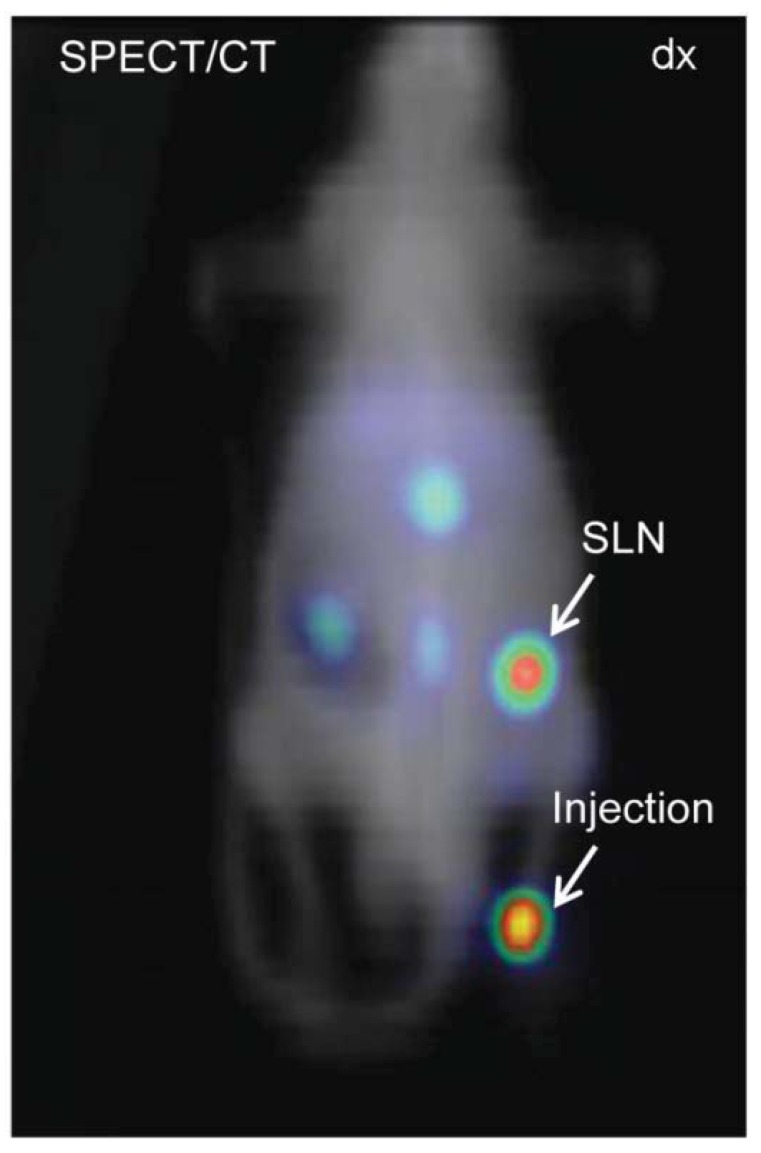
SPECT/CT image of the same animal shown by MR in Figure 2. The arrows depict the injection site and accumulation of the ^99m^Tc-AF-SPIONs in SLN. SPECT is less affected by attenuation compared with optical imaging, therefore, is an invaluable tool to quantify and study the biodistribution of the newly developed agent.

The SPECT/CT images were transferred to a workstation where they were reconstructed and analyzed (Xeleris Functional Imaging Workstation, GE Healthcare) and an in-house developed data visualization software (Interactive Data Language, IDL) was used. The representative image clearly visualized the injection site and inguinal lymph node (right side) Figure 3. Small amounts of the ^99m^Tc-AF-SPIONs could also be detected bilaterally in the inguinal lymph node (left side, second node) as well as in the renal (third) lymph node, 5 h post injection. 

#### 3.3.3. Optical Imaging

**Figure 4:**
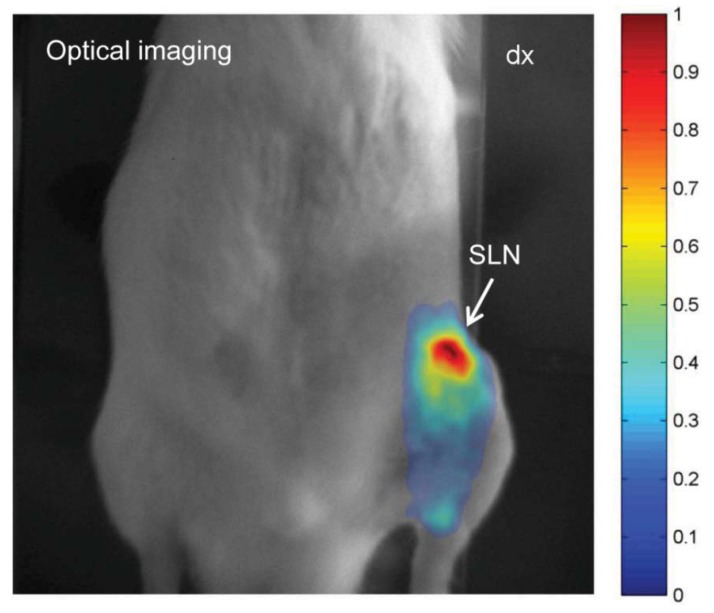
Optical fluorescence image visualizing the SLN. High signal to background ratio is demonstrated which encourage for possible translation of this approach to clinical applications.

**Figure 5:**
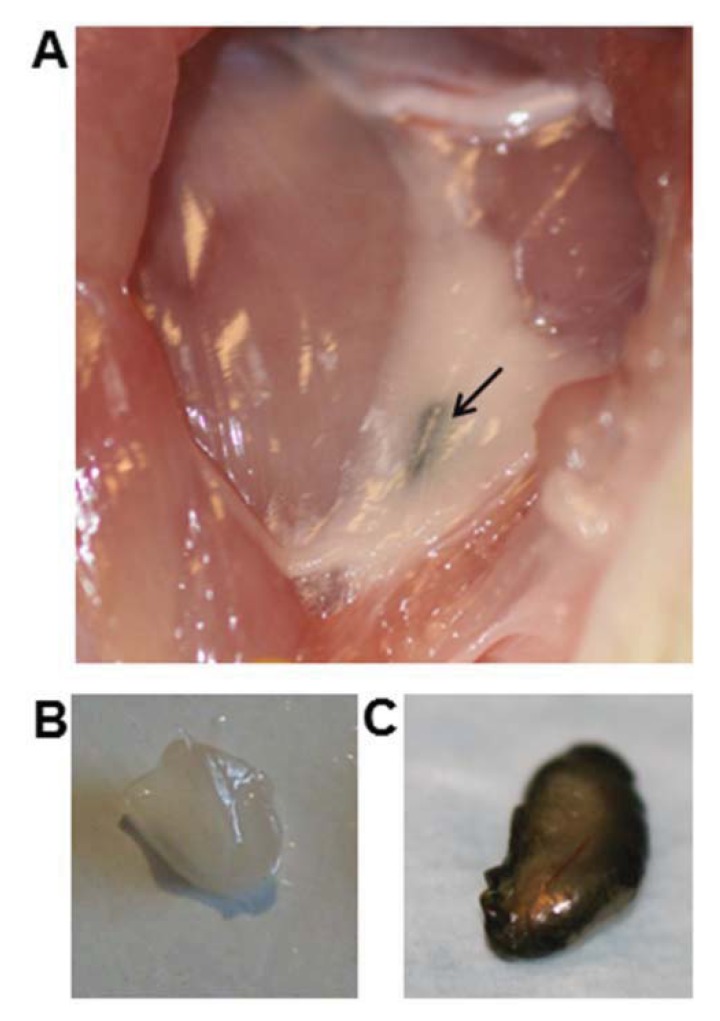
Intraoperative identification of the SLN using ^99m^Tc-AF-SPIONs. (**a**) Similar to the clinical procedure using blue dye, ^99m^Tc-AF-SPIONs stain the SLN green, which makes it easy to be identified during surgery. (**b**) The reference node from the collateral side of the animal. (**c**) The resected SLN.

Figure 4 displays an image of the fluorescence signal originating from the ^99m^Tc–Alexa Fluor 647 labeled SPIONs. An accumulation of the fluorescence signal was observed and its location matches that of the inguinal lymph node. The injection site was not within the imaged region. Lymph nodes deeper than 2 cm have not been detected. However, intraoperatively, the agent showed to be useful to identify the SLN. ^99m^Tc-AF-SPIONs stains the SLN green (Figure 5) and can provide a real time visual guidance during surgery just like methylene blue administrated to patients in the clinical procedure [35,36]. In addition, ^99m^Tc-AF-SPIONs can be administrated in a single injection, compared with the world-wide accepted SLN standard procedure, injecting the radiocolloids and dye separately.

**Figure 6:**
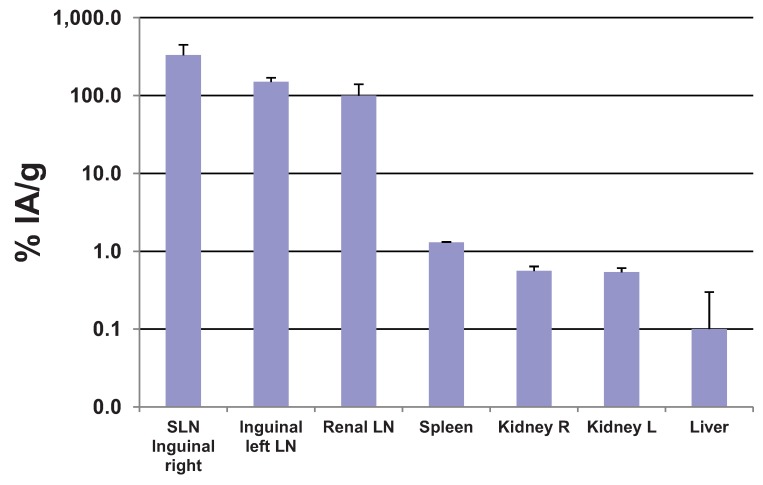
Biodistribution of ^99m^Tc-AF-SPIONs in two white Wistar rats, 5 h post injection.

**Figure 7:**
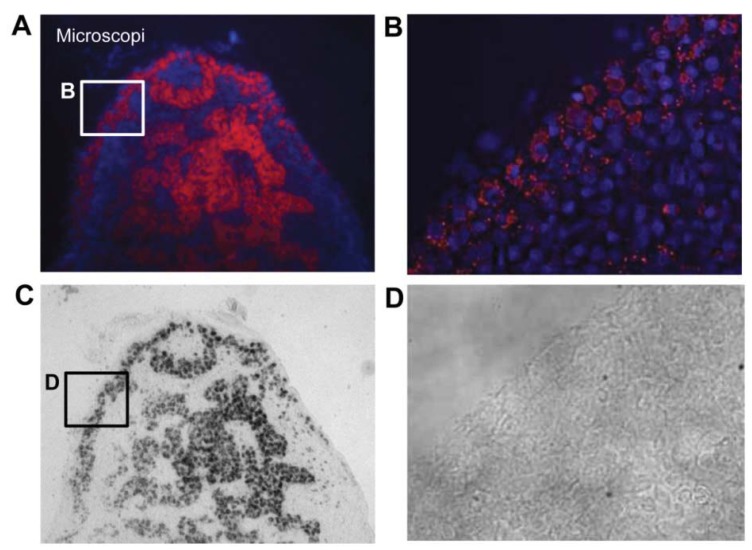
Microscopy images of a cryosectioned SLN. (**a**) Coronal section (20 µm) of a half SLN indicating the microdistribution of the nanoparticles within the SLN. The blue structures are cell nucleus and the red light is emitted from the ^99m^Tc-AF-SPIONs. The nanoparticles accumulate in the cortex and within the medullary sinuses. (**b**) Image visualizing the cortex of the SLN and indicating that the nanoparticles are mostly located extracellularly in comparison with the medullary sinus where the nanoparticles seem to be located within the macrophages. (**c**) and (**d**) bright field images of the SLN corresponding to (**a**) respectively (**b**) which show the orientation and anatomy.

### 3.4. Biodistribution

The uptake of the ^99m^Tc-AF-SPIONs within lymph nodes (LN) was confirmed by the biodistribution analysis. The highest mean uptake was found in the inguinal LN (right side), 330 ± 120% IA/g, followed by the inguinal left side (150 ± 20% IA/g), renal LN side (100 ± 40% IA/g), spleen (1.3 ± 0.02% IA/g), kidneys (0.56 ± 0.08% IA/g) and liver (0.1 ± 0.2% IA/g) as presented in Figure 6. These results suggest a higher uptake of the ^99m^Tc-AF-SPIONs in SLN compared with our previous study using ^99m^Tc-SPIONs (211 ± 225% IA/g) [28]. The higher uptake is often correlated with higher accuracy in localization of SLN. 

Digital autoradiography and microscopy showed that ^99m^Tc-AF-SPIONs indeed localized to SLN. The fluorescence dye enabled localization and studies of microdistribution of the nanoparticles when the ^99m^Tc radioactivity was no longer measurable. Furthermore, a non-homogenous uptake of the nanoparticles was found within the SLN as shown in (Figure 7). The nanoparticles were found both extracellularly and intracellular within the cortex and in sinuses. 

## 4. Conclusions

In conclusion, we developed a new triple-modality imaging agent, ^99m^Tc-AF-SPIONs, for MR, SPECT and optical fluorescence imaging. The easy and straight forward labeling procedure and stability of the agent can be used for detection and intra-operative localization of the SLNs. 
